# TLE1 Positive Clear Cell Sarcoma of the Kidney: A Case Report and Review of the Literature

**DOI:** 10.1155/2018/3462096

**Published:** 2018-10-16

**Authors:** Rana Naous

**Affiliations:** Department of Pathology, SUNY Upstate Medical University, 750 East Adams Street, Syracuse, NY 13210, USA

## Abstract

Clear cell sarcoma of the kidney (CCSK) is an uncommon malignant tumor of uncertain histogenesis that occurs most commonly in childhood. Histologically, CCSK can mimic myxoid variant of synovial sarcoma (SS); however, the double positivity for CD99 and TLE1 in SS helps in excluding CCSK. Herein, we report a rare case of CCSK arising in the left kidney of a 3-year-old girl. The mass grossly measured 9.5 cm in maximum dimension and histologically showed the classic arborizing fibrovascular septae and background myxoid matrix that is usually seen in CCSK. The tumor however was double positive for CD99 and TLE1 which made it difficult to discriminate it from the myxoid variant of SS based on histopathological examination and immunophenotype alone, and genetic analysis for SYT gene rearrangement was required to reach a definitive diagnosis. Although there have been previous case reports of CCSK with positive expression of CD99 and negative TLE1, to our knowledge, this is the first case of CCSK expressing both CD99 and TLE1.

## 1. Introduction

Clear cell sarcoma of the kidney (CCSK) is an uncommon malignant tumor of uncertain histogenesis that occurs most commonly in childhood [1]. Only rare cases have been reported in adults [2]. It represents 4-5% of primary renal neoplasms and is known for its aggressive behavior, tendency for recurrence, and metastasis to bone [3–6]. It is also the second most common pediatric renal tumor after Wilms' tumor [7, 8]. Histologically, CCSK can mimic myxoid variant of synovial sarcoma (SS); however, the double positivity for CD99 and TLE1 in SS helps in excluding CCSK. Herein, we report a rare case of CCSK with double positivity for CD99 and TLE1 whereby it was difficult to discriminate CCSK from the myxoid variant of SS based on histopathological examination and immunophenotype alone, and genetic analysis for SYT gene rearrangement was required to reach a definitive diagnosis. Although there have been previous case reports of CCSK with positive expression of CD99 and negative TLE1, to our knowledge, this is the first case of CCSK expressing both CD99 and TLE1.

## 2. Case Presentation

**Figure 1:**
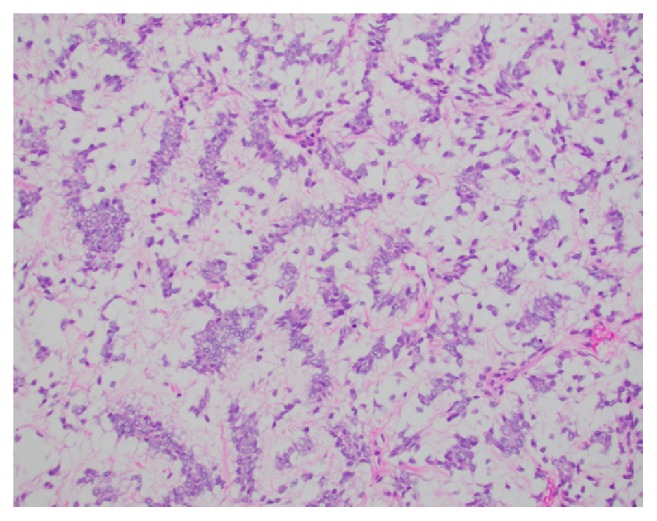
The neoplastic tumor cells are arranged in nests within a myxoid background (H&E, 200x).

**Figure 2:**
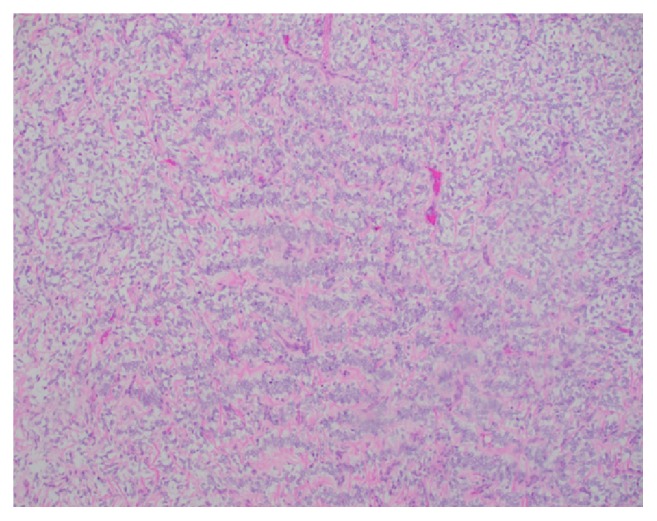
Focal areas with palisading are identified within the tumor (H&E, 100x).

**Figure 3:**
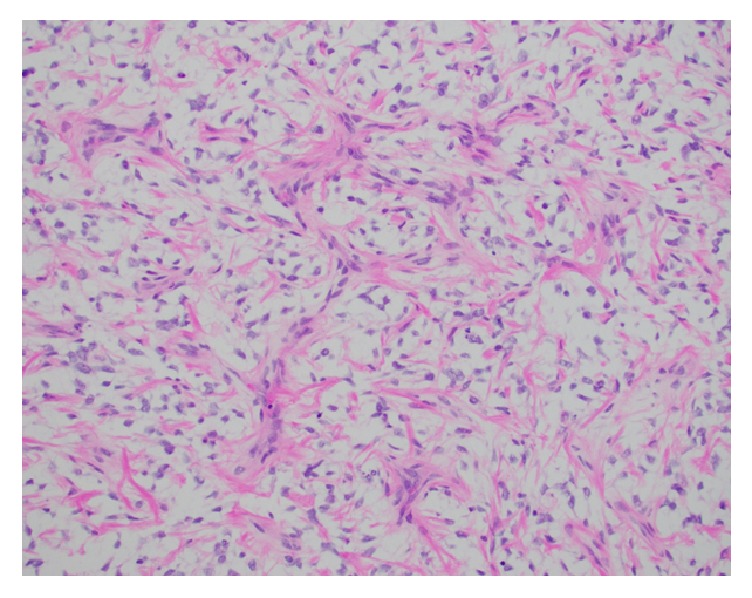
The arborizing fibrovascular septae classic of CCSK are easily seen (H&E, 200x).

**Figure 4:**
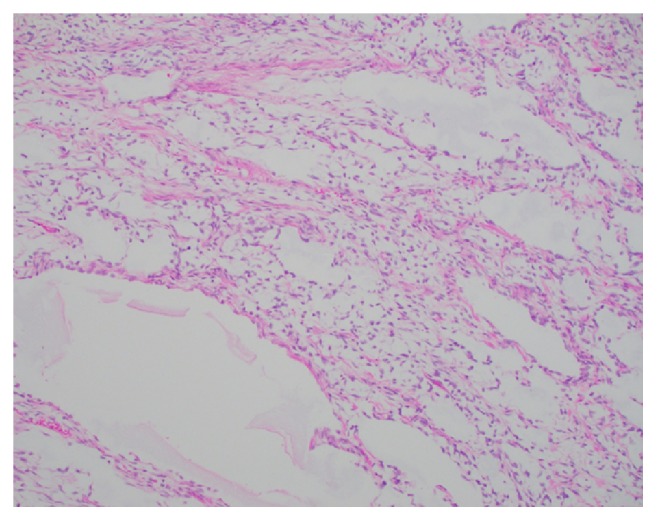
Myxoid pool formation is noted occasionally throughout the tumor (H&E, 200x).

**Figure 5:**
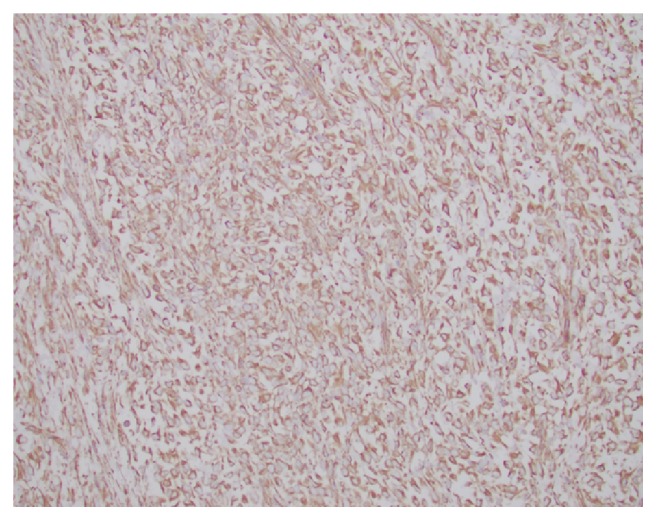
The tumor cells are strongly and diffusely positive for vimentin.

**Figure 6:**
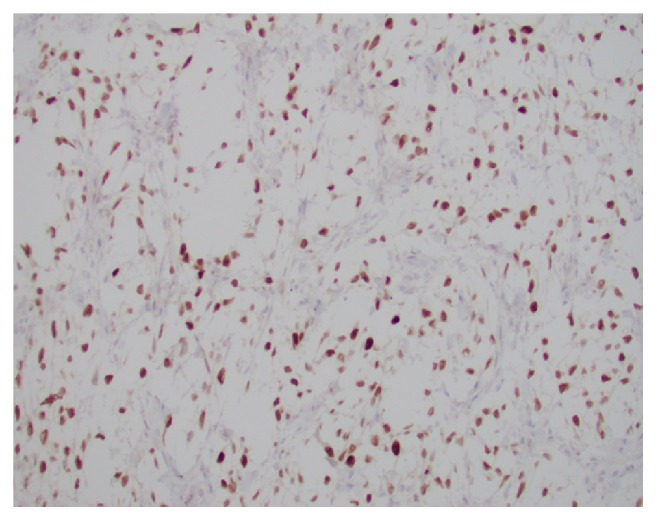
Positive nuclear staining for Cyclin D1 in the tumor cells.

**Figure 7:**
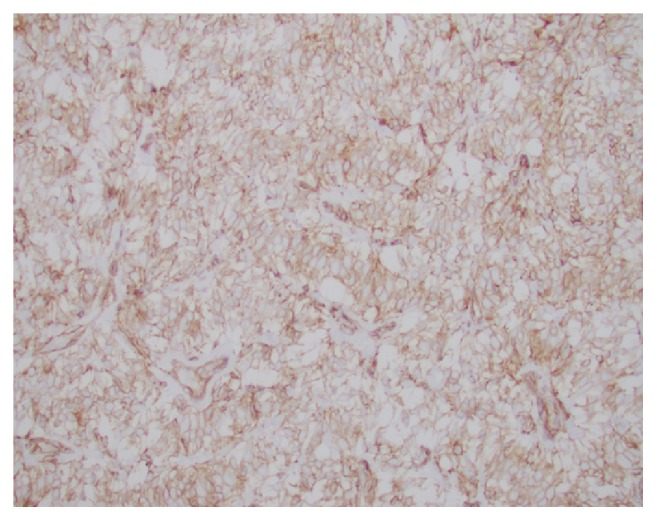
CD99 is diffusely and strongly staining the tumor cells in a membranous pattern.

**Figure 8:**
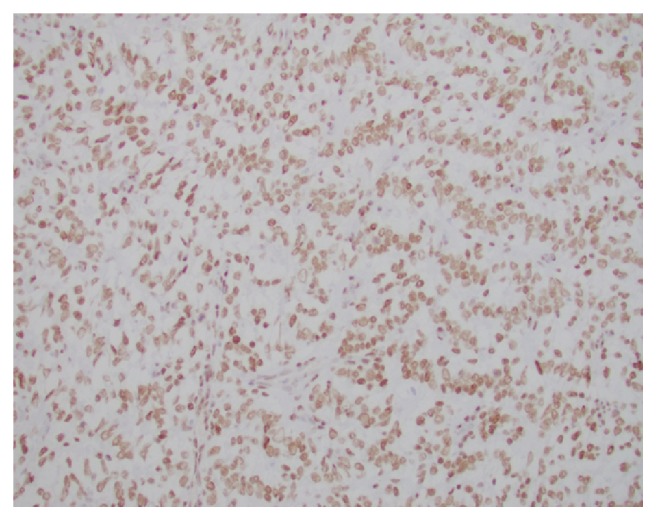
TLE1 has strong and diffuse nuclear positivity in the tumor cells.

**Figure 9:**
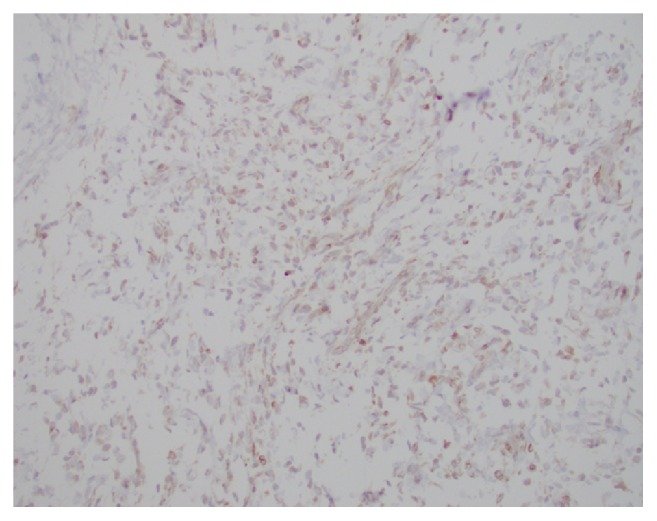
BCL-2 is highlighting focal areas within the tumor.

**Figure 10:**
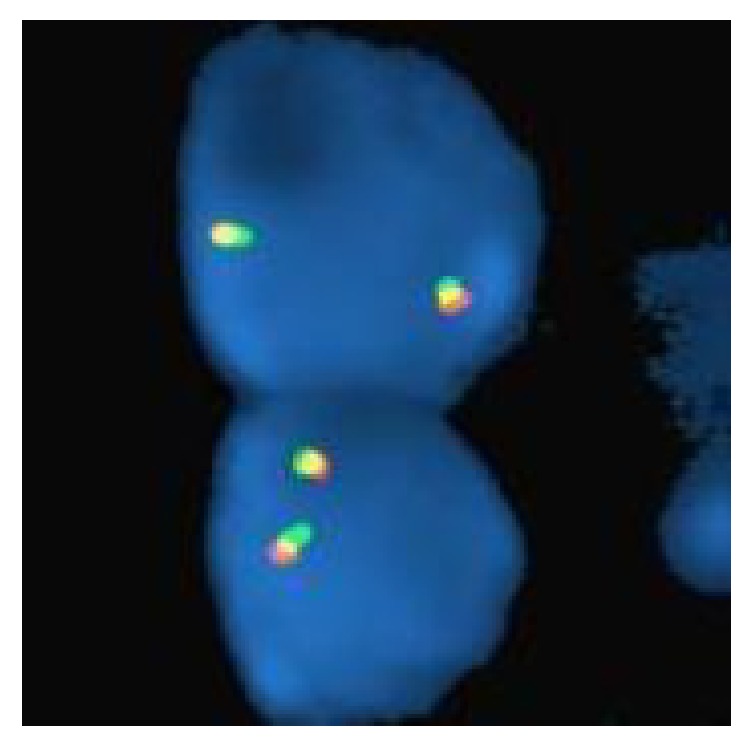
Absent* SS18 (SYT) *gene break-apart rearrangement on chromosome 18q11.2 by FISH (fluorescence in situ hybridization).

A 3-year-old girl presented to the emergency department with eye pain and was found to be hypertensive with a blood pressure measurement of 162/126. Further workup with renal ultrasound demonstrated a heterogeneous mass measuring 9.5 x 9.1 x 8.6 cm occupying the location of the left renal fossa. Surgical resection of the left renal mass revealed a 577.9 gram, 12.0 x 10.2 x 8.0 cm grossly distorted kidney with a 12.0 x 10.0 x 8.3 cm encapsulated, fleshy, pink-gray lesion which appeared grossly to have replaced the majority of the renal parenchyma. Microscopic examination revealed a cellular proliferation of neoplastic cells arranged haphazardly, in cords (Figure 1), occasional nests, and focally palisading (Figure 2) and separated by regularly spaced arborizing fibrovascular septa within an extracellular myxoid matrix (Figure 3) with occasional myxoid pool formation (Figure 4). Necrotic foci were noted focally within the tumor. Immunohistochemical stains were positive for vimentin (Figure 5), cyclin D1 (Figure 6), CD99 (Figure 7), TLE1 (Figure 8), and focally positive for Bcl- 2 (Figure 9) in the tumor cells. SMA, desmin, CD34, cytokeratin AE1/AE3, EMA, WT-1, myogenin, and S100 were negative. The overall morphology and immunopositivity for vimentin, Bcl-2, and cyclinD1 were suggestive of clear cell sarcoma of the kidney. However, given the histologic findings and the tumor immunopositivity for CD99 and TLE1, myxoid variant of synovial sarcoma entered the differential diagnosis. FISH for SYT gene rearrangement (Figure 10) was performed and was negative, ruling out a synovial sarcoma. The final diagnosis was clear cell sarcoma of the kidney, COG Stage III.

## 3. Discussion

Mirkovic et al. have demonstrated in their study that Cyclin D1 is a sensitive marker for CCSK [9, 10]. SATB2 [11], vimentin, and Bcl-2 are also well recognized immunostains that often label CCSK, while other immunomarkers such asTLE1, CD34, S100, desmin, CD99, and cytokeratin are often reported to be negative [12, 13]. Additionally, TLE1 immunostain had not been previously studied in CCSK.

A clinicopathologic study preformed by He L. et al. [14] on 45 pediatric cases of CCSK showed the classic arborizing fibrovascular stroma in all the CCSK cases with variable myxoid, spindle, palisading, epithelioid, sclerosing, cellular, cystic, and angiectatic change. Immunohistochemically, all cases were positive for vimentin but negative for CD99, EMA, CK, desmin, actin, S-100, NSE, CD34, and LCA. TLE1 immunostain was not performed by the authors.

A review of 351 cases of CCSK from the National Wilms Tumor Study Group Pathology Center by Argani P. et al. [15] whereby immunohistochemical stains were performed on 45 out of the 351 cases showed that only vimentin was consistently immunoreactive in all the 45 CCSK cases, while CD99 was consistently negative and TLE1 immunostain was never performed.

It is well known that primary renal synovial sarcomas also express CD99, cyclin D1, and TLE1 which creates a potential overlap with CCSK in some cases. Usually, genetic analysis for SS18 -SSX gene fusions helps in resolving the differential diagnosis of CCSK and primary renal synovial sarcoma.

Hirose M. et al. [16] reported a case of CCSK that was positive for CD-99, vimentin, Bcl-2, and CD-56, and negative for TLE1. Their differential diagnosis suggested CCSK or SSK; however, a final diagnosis of spindle cell pattern CCSK was made based on the absence of the SYT-SSX fusion gene by polymerase chain reaction.

At the genetic level, the majority of CCSKs have internal tandem duplications (ITDs) of the BCOR gene, whereas a minority has the YWHAE-NUTM2 or YWHAE-FAM22 [9] gene fusion, and a third category [17] comprises CCSKs with double negativity for BCOR ITDs, YWHAE-NUTM2, and YWHAE-FAM22 fusion.

Argani P. et al. [18] reported 2 primary renal sarcomas demonstrating BCOR-CCNB3 gene fusions with histologic overlap with CCSK and positive immunoreactivity for BCOR, cyclin D1, TLE1, and SATB2 in the neoplastic cells. They concluded that renal sarcomas with BCOR-CCNB3 gene fusion overlap with CCSK and are in keeping with a “BCOR alteration family” of renal and extrarenal neoplasms which includes CCSK and undifferentiated round cell sarcomas of soft tissue and bone/soft tissue sarcomas with BCOR-CCNB3 gene fusion, all of which are driven by BCOR overexpression and have overlapping clinicopathologic features. Although both cases in Argani's article were positive for BCOR, TLE1, cyclin D1, and SATB2 immunostains, and TLE1 was also positive in the typical CCSK in their control group, they were negative for CD99, desmin, cytokeratin, S100, and CD34 in the tumor cells. This is in contrast to our case that labeled positive for both CD99 and TLE1, and thus marking it as the first case of CCSK to have double positivity for these two markers.

TLE1 or “transducin-like enhancer of split 1,” is one of 4* TLE* genes [19] that is located at chromosome 9q21.32 [20]. It is a transcriptional corepressor that affects signaling pathways and is also involved in modulating differentiation through inhibition of the Wnt / beta catenin signaling cascade [21].

TLE1 was previously regarded as both highly sensitive and specific for synovial sarcoma with expected intense, diffuse nuclear staining in the tumor cells [22]. However, further studies have shown its positivity in many other nonsynovial sarcoma entities including endometrial stromal sarcoma which has been reported to manifest limited TLE1 immunoreactivity [23]. TLE1 immunoreactivity has also been demonstrated in soft tissue or bone sarcomas with BCOR-CCNB3 gene fusion [24]. Given all that, we are uncertain of the mechanism behind the TLE1 immunopositivity in our CCSK case; however, we postulate that the presence of the YWHAE-FAM22 rearrangement, identical to that in endometrial stromal sarcoma, in a minority of CCSK cases [9] or the recent demonstration of BCOR-CCNB3 gene fusions [18] in rare cases of CCSK may play a role in this finding.

In conclusion, we report a rare case of CCSK with double positivity for CD99 and TLE1 whereby it was difficult to discriminate CCSK from the myxoid variant of SS based on histopathological examination and immunophenotype alone, and genetic analysis for SYT gene rearrangement was required to reach a definitive diagnosis. Our case adds to the list of non-SS entities with TLE1 immunopositivity and emphasizes the role of genetic testing as a more specific method of diagnosis.
